# H_2_ Protects Against Lipopolysaccharide-Induced Cardiac Dysfunction *via* Blocking TLR4-Mediated Cytokines Expression

**DOI:** 10.3389/fphar.2019.00865

**Published:** 2019-08-05

**Authors:** Sihua Tan, Zhiyuan Long, Xiangping Hou, Yujie Lin, Jingting Xu, Xinchao You, Tinghuai Wang, Yaxing Zhang

**Affiliations:** ^1^Department of Physiology, Zhongshan School of Medicine, Sun Yat-sen University, Guangzhou, China; ^2^Department of Psychological Sleeping, Second School of Clinical Medicine, Guangzhou University of Chinese Medicine, Guangzhou, China; ^3^Department of Traditional Chinese Medicine, Sun Yat-sen Memorial Hospital, Sun Yat-sen University, Guangzhou, China; ^4^Biofeedback Laboratory, Xinhua College of Sun Yat-sen University, Guangzhou, China; ^5^Department of Biomedical Engineering, Xinhua College of Sun Yat-sen University, Guangzhou, China; ^6^Department of Science and Education, The First Affiliated Hospital/School of Clinical Medicine of Guangdong Pharmaceutical University, Guangzhou, China; ^7^Biofeedback Therapy and Research Laboratory, Sun Yat-sen Memorial Hospital, Sun Yat-sen University, Guangzhou, China; ^8^Department of Traditional Chinese Medicine, The Third Affiliated Hospital, Sun Yat-sen University, Guangzhou, China

**Keywords:** hydrogen gas, septic cardiomyopathy, TLR4, TNFα, IL-1β, IL-18

## Abstract

**Background and Purpose:** Septic cardiomyopathy, which is one of the features of multi-organ dysfunction in sepsis, is characterized by ventricular dilatation, reduced ventricular contractility, and reduction in ejection fraction and, if severe, can lead to death. To date, there is no specific therapy that exists, and its treatment represents a large unmet clinical need. Herein, we investigated the effects and underlying anti-inflammatory mechanisms of hydrogen gas in the setting of lipopolysaccharide (LPS)-induced cardiomyocytes injury.

**Experimental Approach:** Hydrogen gas was intraperitoneally injected to mice in LPS plus hydrogen group and hydrogen group for 4 days. On fourth, LPS was given by intraperitoneal injection to mice in LPS group and to mice in LPS plus hydrogen group. In addition, H9c2 cardiomyocytes were treated with hydrogen-rich medium for 30 min before LPS. The transthoracic echocardiography was performed at 6 h post‐LPS to assess left ventricular end-systolic diameter (LVESD), left ventricular end-diastolic diameter (LVEDD), left ventricular ejection fraction (EF%), fractional shortening (FS%), left ventricular mass average weight (LV mass AW), and LV mass AW (Corrected). The histological and morphological analyses of left ventricular were performed by hematoxylin and eosin (H&E) staining and Masson’s trichrome staining. The mRNA levels of ANP and BNP were examined by PCR *in vitro*. The expression of cytokines were assayed by Enzyme Linked Immunosorbent Assay (ELISA) and PCR. Moreover, Western blotting was performed to examine the expression of TLR4, the activation of ERK1/2, p38, JNK, and the expression of NF-κB in nucleus after 6 h of LPS challenge *in vivo* and *in vitro*.

**Key Results:** LPS induced cardiac dysfunction; hydrogen therapy improved cardiac function after LPS challenge. Furthermore, pretreatment with hydrogen resulted in cardioprotection during septic cardiomyopathy *via* inhibiting the expression of pro-inflammatory cytokines TNFα, IL-1β, and IL-18; suppressing the phosphorylation of ERK1/2, p38, and JNK; and reducing the nuclear translocation of NF-κB and the expression of TLR4 by LPS.

**Conclusion and Implications:** Hydrogen therapy prevents LPS-induced cardiac dysfunction in part *via* downregulation of TLR4-mediated pro-inflammatory cytokines expression.

## Introduction

Sepsis is a clinical syndrome, affecting ∼19 million individuals per year worldwide, characterized by a maladaptive host response with ensuing life-threatening organ dysfunction resulting from infection (Reinhart et al., 2017; Weis et al., 2017). Cardiac involvement frequently complicates to sepsis, which, if severe, can lead to death (Havaldar, 2018; Martin et al., 2019; Ward and Fattahi, 2019). Characteristically, early studies indicate cardiac dysfunction in adequately volume-resuscitated septic patients with increased end-diastolic volume index and decreased ejection fraction, and these acute changes in ejection fraction and end-diastolic volume index, although sustained for several days, were reversible (Weisel et al., 1977; Calvin et al., 1981; Parker et al., 1984). More recently, studies in cellular levels, isolated heart studies, animal models *in vivo*, and in human studies, have clearly established decreased contractility and impaired myocardial compliance as major factors that cause myocardial dysfunction in sepsis (Merx and Weber, 2007; Martin et al., 2019).

The bacterial endotoxin lipopolysaccharide (LPS), which is a component of the outer membrane of Gram-negative bacteremia, has been regarded as a main culprit responsible for cardiac dysfunction in sepsis (Bai et al., 2016). Mechanistically, LPS associates with its receptor toll-like receptor 4 (TLR4) through the help of LPS-binding protein CD14 and, subsequently, results in the production of inflammatory cytokines, such as TNFα, IL-1β, and IL-18, which might directly disturb cardiac function (Knuefermann et al., 2002; Raeburn et al., 2002; Zhang et al., 2017a). Although tremendous efforts have been made during the last decades, no specific therapy for sepsis-induced cardiomyopathy exists (Weis et al., 2017). Therefore, there is urgent need of innovative therapeutic options for sepsis-induced cardiomyopathy, which is essential for reducing the mortality of sepsis.

Hydrogen gas (H_2_), a medical gas that has anti-oxidant, anti-apoptotic, and anti-inflammatory properties (Ohsawa et al., 2007; Yu et al., 2011; Zhai et al., 2013; Zhai et al., 2014; Li et al., 2016; Zhai et al., 2017), has been reported to improve sepsis-induced organ dysfunction, such as lung (Qiu et al., 2011; Liang et al., 2012; Xie et al., 2012; Hattori et al., 2015; Liu et al., 2015; Dong et al., 2017; Dong et al., 2018), liver (Sun et al., 2011; Iketani et al., 2017), and bowel (Sakata et al., 2017). Recent studies have also indicated that H_2_ has strong cardiovascular activities (Zhang et al., 2018). For example, intraperitoneal injection of H_2_ can improve isoproterenol (ISO)-induced cardiac hypertrophy *in vivo* (Zhang et al., 2016a; Zhang et al., 2017b) and inhibit vascular hypertrophy induced by abdominal aortic coarctation *in vivo* (Zhang et al., 2016b). H_2_-rich medium suppresses ISO-induced H9c2 cardiomyocytes hypertrophy and angiotensin II-induced vascular smooth muscle cells proliferation and migration *in vitro* (Zhang et al., 2016b). However, the effects of H_2_ intraperitoneal injection on sepsis-induced cardiomyopathy and the molecular mechanisms remain unclear. The aims of this study are, therefore, to determine the effect of H_2_ intraperitoneal injection on LPS-induced cardiac dysfunction *in vivo* and the effect of H_2_-rich medium on LPS-induced H9c2 cardiomyocytes injury *in vitro*, as well as to identify the molecular mechanisms that may be involved in this process.

## Materials and Methods

### Drugs, Antibodies, and ELISA Kits

LPS (cat no. L2880, Sigma-Aldrich; MerekKGaA, Darmstadt, Germany) was dissolved in normal saline (1 mg/ml), under sterile conditions immediately prior intraperitoneal injection, and dissolved in double distilled water (1 mg/ml) as for cell culture study. H_2_ (99.999%; Guang Zhou Guang Qi Gas Co., Ltd., Guangzhou, China) was stored in a seamless steel gas cylinder. H_2_ was injected into a vacuumed aseptic soft plastic infusion bag (100 ml; CR Double-Crane Pharmaceuticals Co., Ltd, Anhui, China) under sterile conditions immediately prior to intraperitoneal injection for animal study. For cell culture study, the same vacuumed bag was injected with 20-ml Dulbecco’s modified Eagle medium (DMEM; Gibco, New York City, NY, USA), which was supplemented with 100 U/ml penicillin/streptomycin (Hyclone); then, the bag with DMEM was bubbling by H_2_ until this bag was full of H_2_ with no dead volume. The bag was maintained at 4°C for >6 h prior to use, and the concentration of H_2_ was measured as our previously described (Zhang et al., 2016b; Zhang et al., 2017b).

The antibodies against extracellular signal-regulated kinase 1/2 (ERK1/2), p-ERK1/2, p38, p-p38, c-Jun NH2-terminal kinase (JNK), p-JNK, NF-κB p65, and Histone H3 and Glyceraldehyde-3-phosphate dehydrogenase (GAPDH) were from Cell Signaling Technology (Danvers, MA, USA). The antibody against TLR4 was from Santa Cruz Biotechnology (Santa Cruz, CA, USA).

The mouse TNFα, IL-1β Enzyme Linked Immunosorbent Assay (ELISA) kits were from R&D Systems (Minneapolis, USA). The mouse IL-18 ELISA kit was from Medical & Biological Laboratories CO., LTD. (Nagoya, Japan).

### Animal Model of LPS-Induced Cardiac Dysfunction and Treatment Protocol

Male C57BL/6J mice (8 to 10 weeks of age) were used in this study. All animals were housed in a temperature-controlled animal facility with a 12-h light–dark cycle and allowed to obtain rodent chow and water *ad libitum*. All animals received humane care in compliance with the Principles of Laboratory Animal Care formulated by the National Society of Medical Research and the Guide for the Care and Use of Laboratory Animals published by the NIH (8^th^ Edition, Revised 2011) (Polhemus et al., 2017). The Institutional Animal Care and Use Committee (Zhongshan School of Medicine, Sun Yat-sen University) approved all animal procedures.

Mice were randomly assigned to four groups: Control (Con) group (*n* = 10), LPS group (*n* = 10), LPS plus H_2_ group (*n* = 10), and H_2_ group (*n* = 10). In LPS plus H_2_ group and H_2_ group, H_2_ (99.999%) was given at the dose of 1 ml per 100 g/day by intraperitoneal injection as our previously described for 4 days (Zhang et al., 2016a; Zhang et al., 2017b). On the fourth, LPS was given to LPS group and LPS plus H_2_ group by intraperitoneal injection (5 mg/1,000g) as previously revealed (Drosatos et al., 2011), Con group was treated with saline. Moreover, H_2_ was given 0.5 h before LPS injection in LPS plus H_2_ group. Transthoracic echocardiography was performed with a Vevo-2100 high-frequency ultrasound system (VisualSonics Inc., Toronto, Canada) to assess left ventricular ejection fraction (EF%), fractional shortening (FS%), left ventricular end-systolic diameter (LVESD), left ventricular end-diastolic diameter (LVEDD), left ventricular mass average weight (LV mass AW), and LV mass AW (Corrected, = 0.8 × LV mass AW) 6 h post-LPS administration as previously described (Devereux et al., 1986; Nemoto et al., 2002; Hayashida et al., 2008; Wang et al., 2016a).

### Cell Culture and Treatment

H_2_-rich medium was prepared as previously described (Zhang et al., 2017b). H9c2 cardiomyocytes were grown in DMEM containing 5.5-mM glucose (Zhang et al., 2017b). Cells were serum starved for 18 h in DMEM containing 1% FBS and then treated with H_2_-rich medium for 30 min before LPS; finally, the medium was added 1 µg/ml LPS. The expression or activation of kinases, NF-κB, TLR4, and cytokines were examined after 6 h of LPS challenge.

### The Enzyme-Linked Immunosorbent Assay

The levels of TNFα, IL-18, and IL-1β in serum of mice were quantified by the commercial ELISA kits following manufacturer’s instructions. The plates were read on a TECAN infinite F200 Plate Reader, measuring absorbance at 450 nm.

### Quantitative Real-Time PCR (qRT-PCR)

Total mRNA was extracted from H9c2 cardiomyocytes using TRIZol reagent (Invitrogen) as previously described (Zhang et al., 2016a). The oligo (dT) primers with the Transcriptor First Strand cDNA Synthesis Kit (PrimeScript^™^ RT Master Mix, Takara) were used to synthesize cDNA. qRT-PCR was performed using SYBR green (SYBR^®^ Premix Ex Taq^™^II, Takara) on a BIO-RAD CFX96 Touch^™^ Real-time PCR Detection System. GAPDH was used as an internal control. The relative expression level of target genes was calculated using the 2^−ΔΔCt^ method. The primers for qRT-PCR are shown in **Table 1**.

**Table 1:** Sequence of primers used for quantitative real-time PCR (qRT-PCR).

Genes	Species	Sequence (5’ → 3’)
**TNFα**	Rat	(Forward)-AAATGGGCTCCCTCTCATCAGTTC (Reverse)-TCTGCTTGGTGGTTTGCTACGAC
**IL-1β**	Rat	(Forward)-TCGTGCTGTCTGACCCATGT (Reverse)-ACAAAGCTCATGGAGAATACCACTT
**IL-18**	Rat	(Forward)-ACGGAGCATAAATGACCAAGTTC (Reverse)-TCTGGGATTCGTTGGCTGTT
**ANP**	Rat	(Forward)-GGGAAGTCAACCCGTCTCA (Reverse)-GGCTCCAATCCTGTCAATCC
**BNP**	Rat	(Forward)-CTCCAGAACAATCCACGATG (Reverse)-ACAGCCCAAGCGACTGACT
**GAPDH**	Rat	(Forward)-CCAAGGTCATCCATGACAACTT (Reverse)-AGGGGCCATCCACAGTCTT

### Histological and Morphological Analyses

Hearts were harvested for observing histological and morphological alterations by hematoxylin and eosin (H&E) staining and Masson’s trichrome staining as previously revealed (Zhang et al., 2016a; Xu et al., 2019).

### Western Blotting

Western blotting was performed as we have previously described (Zhang et al., 2017b). The nuclear protein were isolated by the NE-PER^™^ Nuclear and Cytoplasmic Extraction Reagent Kit (Thermo Fisher Scientific, Waltham, MA, USA). The proteins were transferred to polyvinylidene fluoride membranes (Millipore, Bedford, MA, USA), which were incubated with primary and secondary antibodies by standard techniques. The enhanced chemiluminescence (ChemiDoc XRS+ System, Bio-Rad, Hercules, CA, USA) was used to accomplish immunodetection.

### Statistical Analysis

Data are expressed as mean ± SD. Statistical analysis was performed by one-way analysis of variance (ANOVA) followed by Bonferroni’s *post hoc* test. A value of *P* < 0.05 was considered as significantly different.

## Results

### H_2_ Alleviates LPS-Induced Cardiac Dysfunction

**Figure 1:**
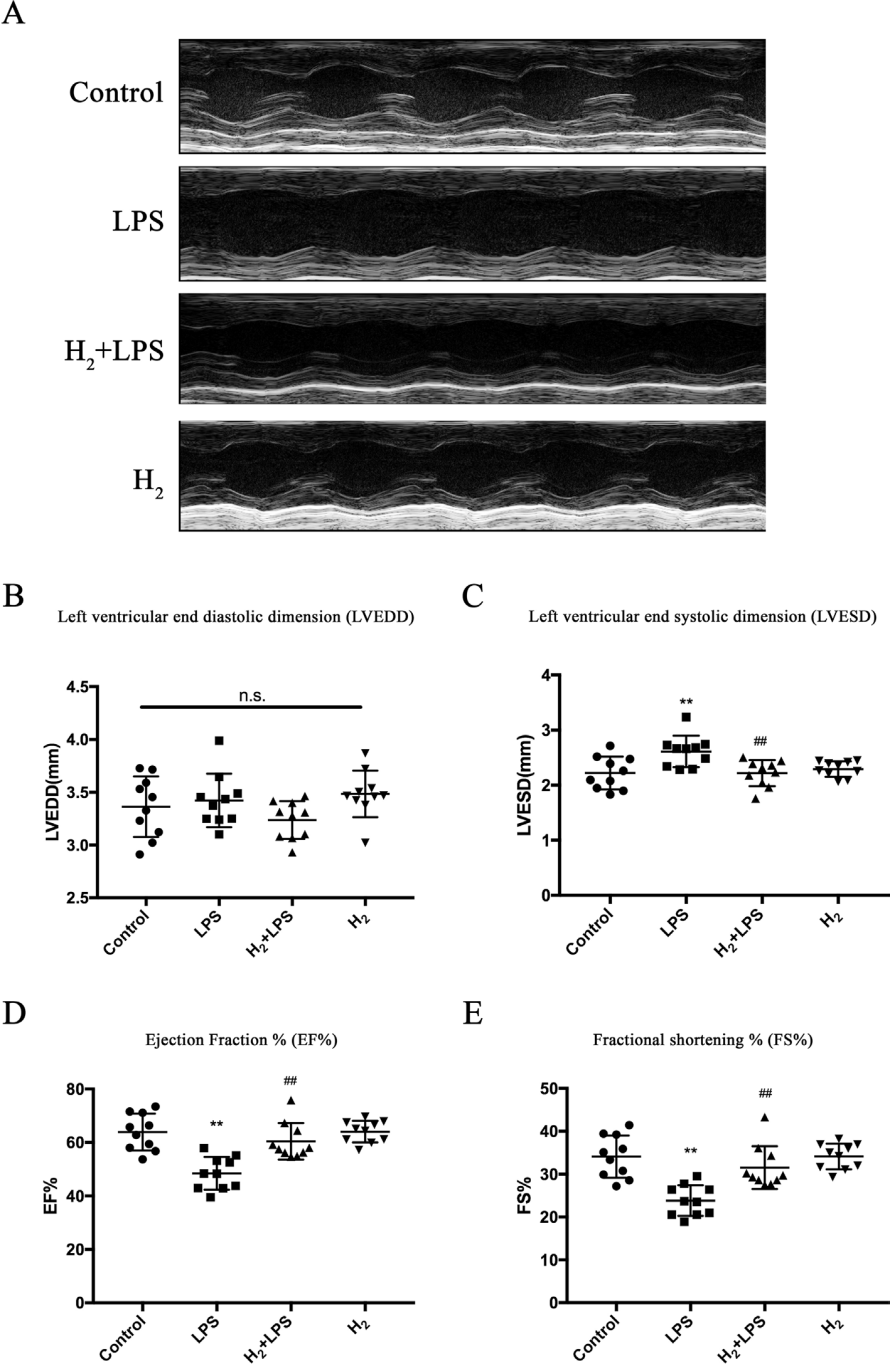
H_2_ protects against lipopolysaccharide (LPS)-induced cardiac dysfunction. **(A)** Representative M-mode echocardiographic images at 6 h after LPS injection. **(B–E)** Quantification of left ventricular end-diastolic diameter (LVEDD), left ventricular end-systolic diameter (LVESD), EF%, and FS% by echocardiography at 6 h after LPS injection (*n* = 10). **P < 0.01 vs Control, ^##^ *P* < 0.01 vs LPS.

We attempted to use intraperitoneal injection of H_2_, to test whether H_2_ can suppress LPS-induced cardiac dysfunction. As previously described (Drosatos et al., 2013), 6 h after LPS challenge, mice exhibited cardiac dysfunction compared with the control group, as indicated by decreasing percent fractional shortening (FS%) and percent ejection fraction (EF%) and increasing LVESD, while left ventricular end-diastolic diameter (LVEDD) was not affected (**Figures 1A–E**). H_2_ injection alleviated the impaired cardiac function by LPS, as evidenced by increasing FS% and EF% and decreasing LVESD (**Figures 1A–E**). Therefore, intraperitoneal injection of H_2_ can improve LPS-induced cardiac dysfunction.

### The Effect of H_2_ on Left Ventricular Structure in the Challenge of LPS

**Figure 2:**
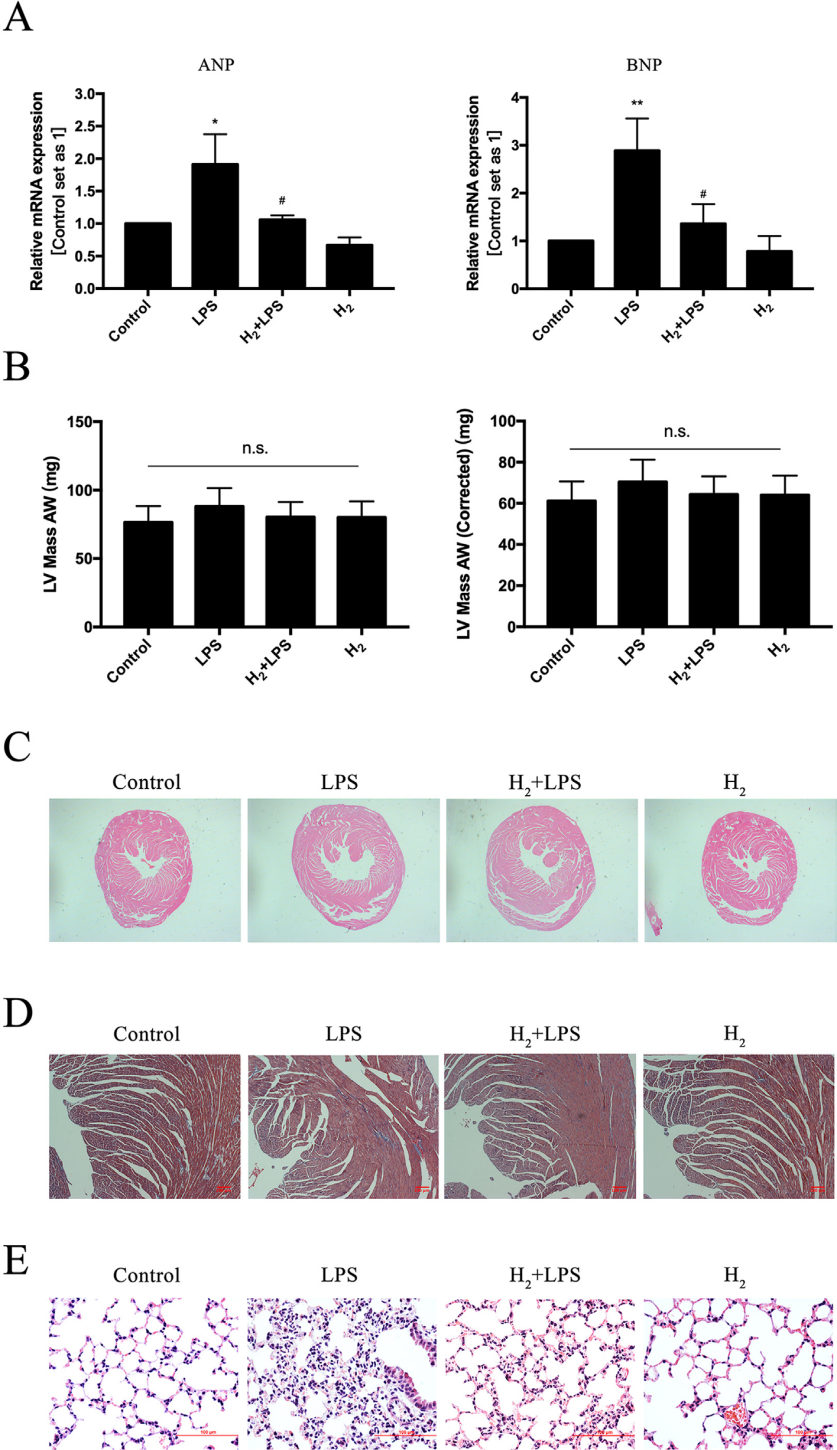
The effect of H_2_ on left ventricular structure in the challenge of LPS. **(A)** Quantification of the mRNA levels of ANP and BNP *in vitro*, GAPDH was used as a loading control (*n* = 3). **(B)** LV mass AW and LV mass AW (Corrected) were calculated by Vevo LAB analysis software from Vevo-2100 high-frequency ultrasound system (*n* = 10). **(C)** Hematoxylin and eosin (H&E) staining for myocardium, 20×. **(D)** Masson’s trichrome staining for myocardium. **(E)** H&E staining for lung. *p < 0.05 vs Control, **P < 0.01 vs Control, ^#^ *P* < 0.05 vs LPS.

LPS can upregulate pathologic hypertrophy marker ANP and BNP and induce cellular hypertrophy in H9c2 cardiomyocytes (Liu et al., 2008). In our study, the upregulation of mRNA levels of ANP and BNP by LPS was suppressed by H_2_-rich medium pretreatment (**Figure 2A**). LPS slightly increased LV mass AW and LV mass AW (Corrected), and the mean of LV mass AW and LV mass AW (Corrected) were lower in H_2_ plus LPS group than that in LPS group, although there was no statistical difference between groups (**Figure 2B**). Similar to the increased in LVESD in LPS group (**Figure 1C**), H&E staining showed that LPS induced left and right ventricular cavities enlargement, decreased the thickness of interventricular septum, and H_2_ improves these phenotype (**Figure 2C**). Moreover, no obvious cardiac fibrosis was induced by LPS, H_2_, and H_2_ plus LPS as determined by Masson’s trichrome staining (**Figure 2D**). As previously discussed (Liang et al., 2012; Xie et al., 2012), the formation of lung edema and the influx of immune-competent cells into the lung tissue by LPS were reduced by H_2_ (**Figure 2E**). Collectively, these data suggested that H_2_ has the potential ability to improve LPS-induced left ventricular structure injury in these mice.

### H_2_ Inhibits LPS-Induced Inflammatory Cytokines Production

**Figure 3:**
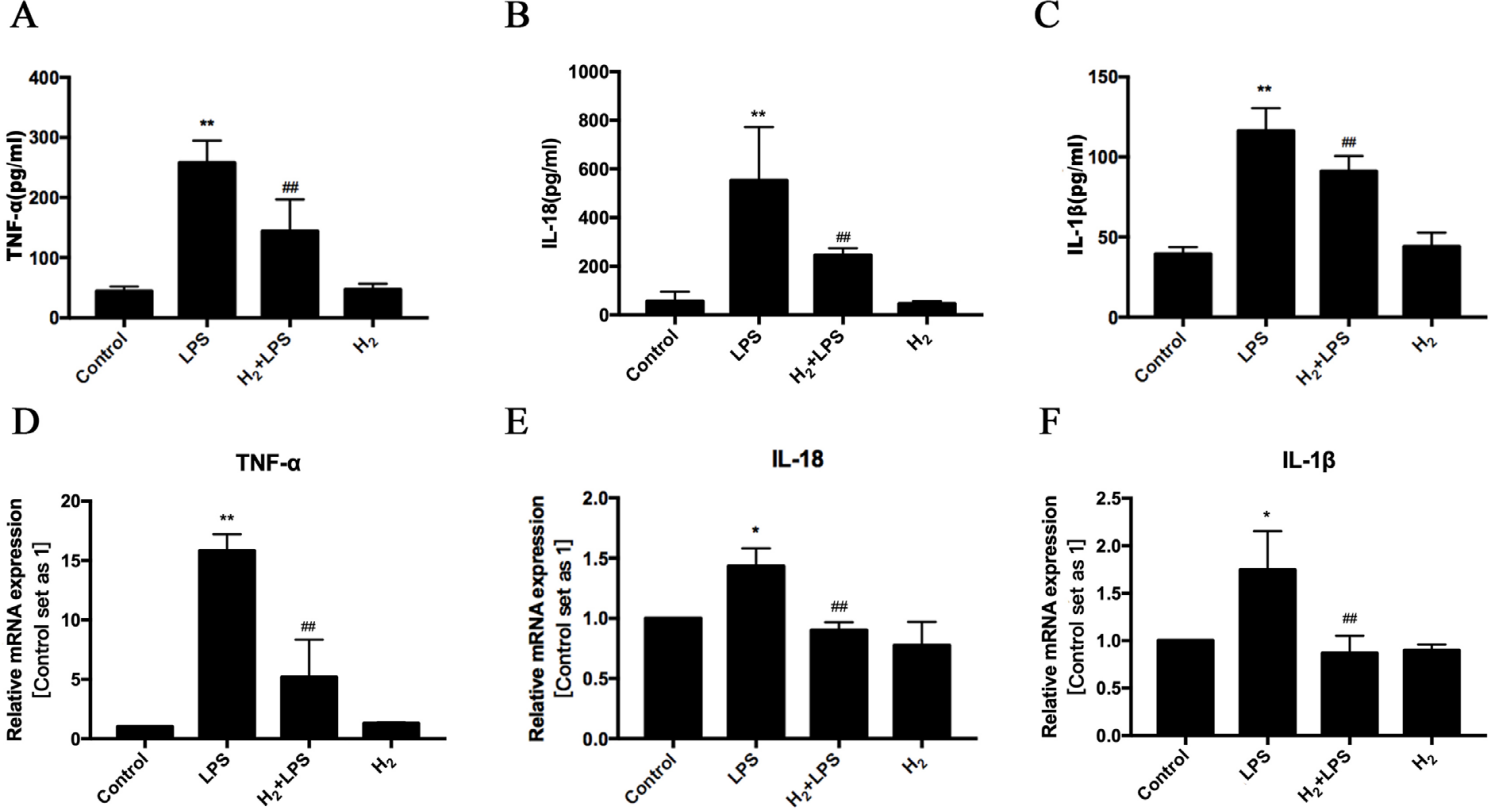
The effects of H_2_ on LPS-induced inflammatory cytokines expression. **(A–C)** The concentration of TNFα, IL-18, and IL-1β in serum (*n* = 5–8). **p < 0.01 vs Control, ^#^ *p* < 0.05 vs LPS, ^##^ *p* < 0.01 vs LPS. **(D–F)** Quantification of the mRNA levels of TNFα, IL-18, and IL-1β in H9c2 cardiomyocytes, GAPDH was used as a loading control (*n* = 3). *p < 0.05 vs Control, **p < 0.01 vs Control, ^##^ *p* < 0.01 vs LPS. n.s. means “no significance.”

As the ligand of pattern recognition receptor (PRR) TLR4, LPS can induce various inflammatory cytokines production (Zhang et al., 2017a). Among these cytokines**,** TNFα, IL-1β, and IL-18 have been reported to result in cardiac dysfunction (Knuefermann et al., 2002; Raeburn et al., 2002; Drosatos et al., 2011; Okuhara et al., 2017). As previous studies (Knuefermann et al., 2002; Raeburn et al., 2002; Drosatos et al., 2011; Drosatos et al., 2013; Okuhara et al., 2017), LPS increases the protein levels of TNFα, IL-1β, and IL-18 in serum (**Figures 3A–C**) and enhanced the mRNA levels of TNFα, IL-1β, and IL-18 in H9c2 cardiomyocytes (**Figures 3D–F**). These increased TNFα, IL-1β, and IL-18 can be suppressed by pretreatment of intraperitoneal injection of H_2_ *in vivo* (**Figures 3A–C**) and pretreatment of H_2_-rich medium *in vitro* (**Figures 3D–F**). Therefore, these results indicate that H_2_ inhibits the expression of inflammatory cytokines in serum and cardiomyocytes during sepsis-induced cardiomyopathy.

### H_2_ suppresses TLR4-Mediated Innate Immune signaling

**Figure 4:**
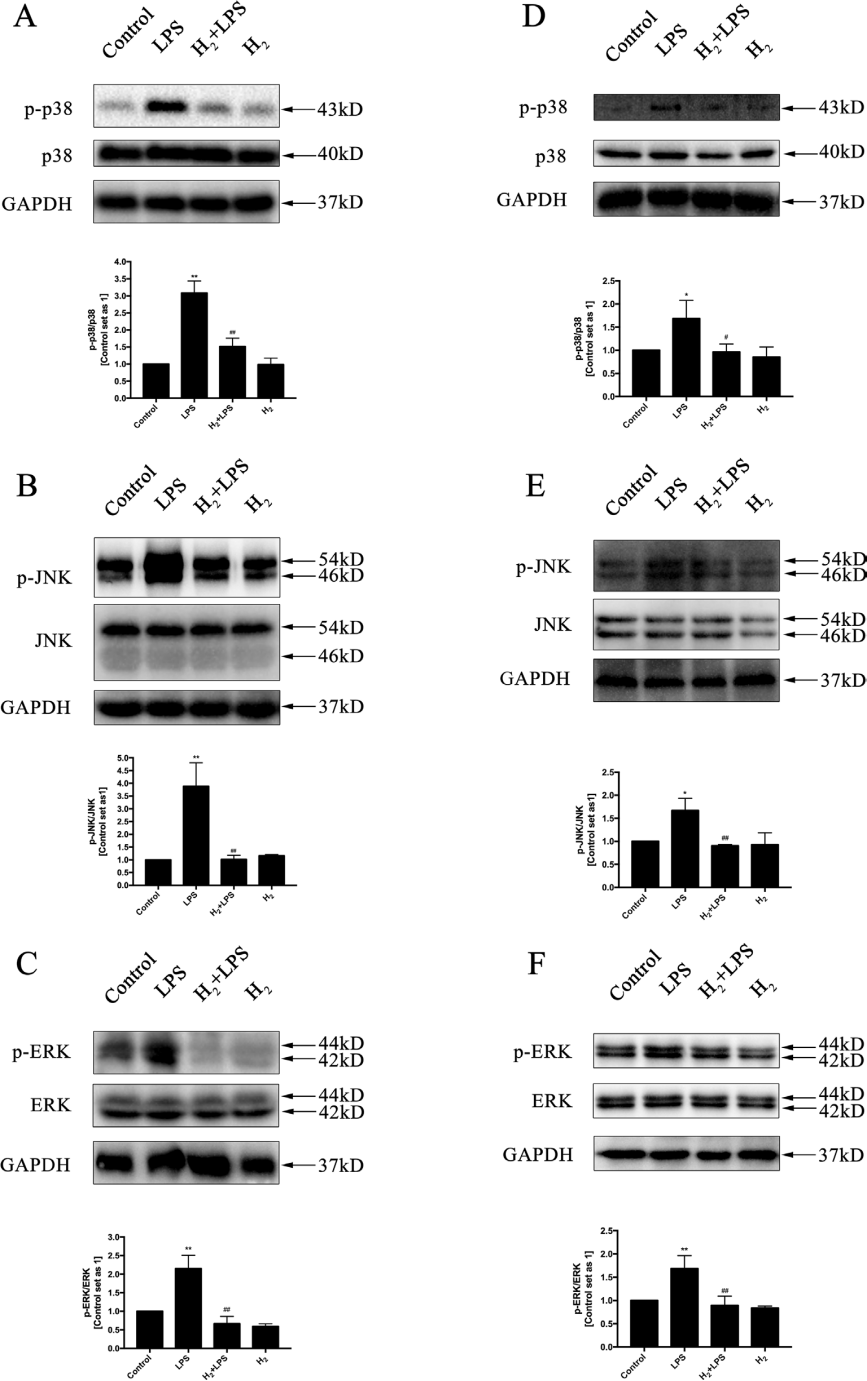
The effects of H_2_ on LPS-mediated MAPK signaling activation. Representative Western blot and quantification of p38 phosphorylation **(A** and **D)**, JNK phosphorylation **(B** and **E)**, and ERK1/2 phosphorylation **(C** and **F)** to their total protein expressions (*n* = 3). *p < 0.05 vs Control, **p < 0.01 vs Control, ^#^ *p* < 0.05 vs LPS, ^##^ *p* < 0.01 vs LPS.

**Figure 5:**
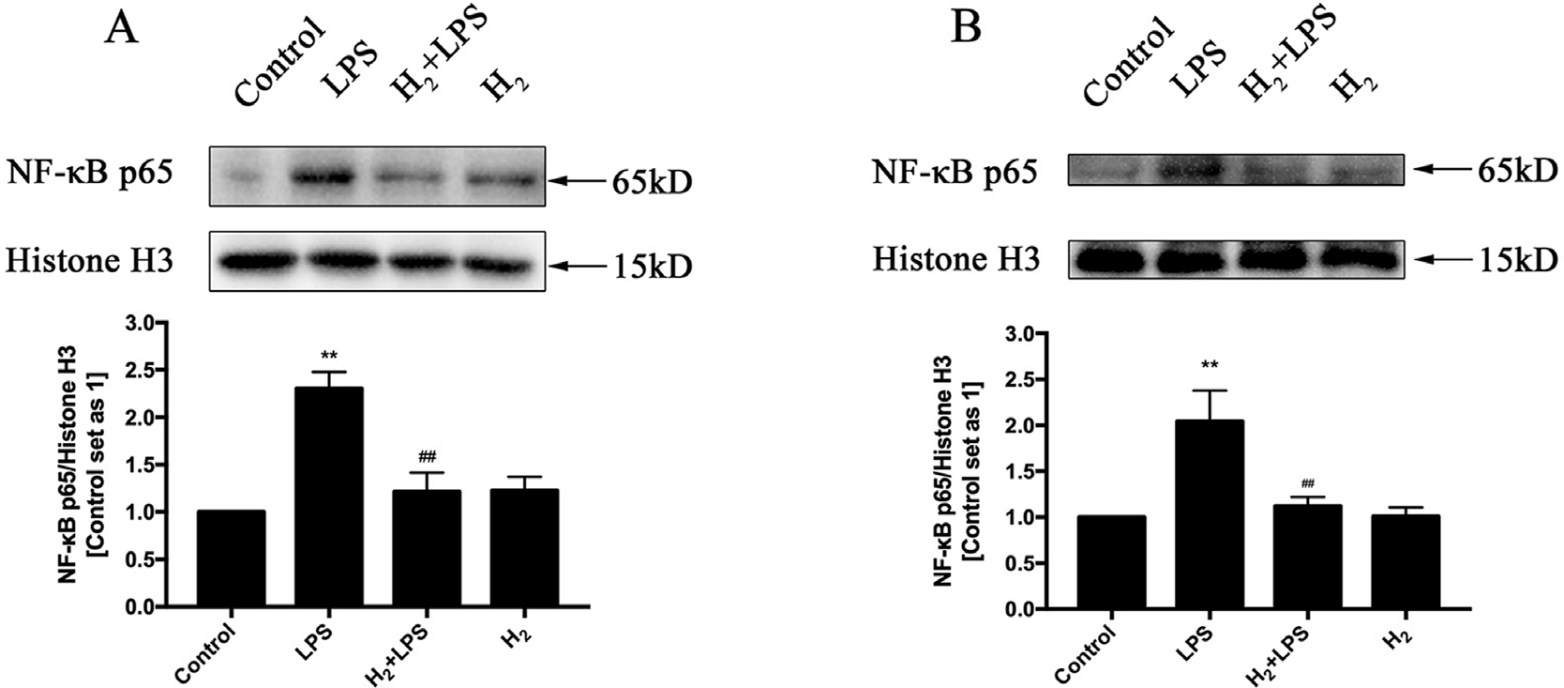
The effects of H_2_ on LPS-mediated nuclear translocation of NF-κB. Representative Western blot and quantification of nuclear NF-κB to Histone H3 *in vivo* **(A)** and *in vitro* **(B)**, respectively (*n* = 4). **p < 0.01 vs Control, ^##^ *p* < 0.01 vs LPS.

**Figure 6:**
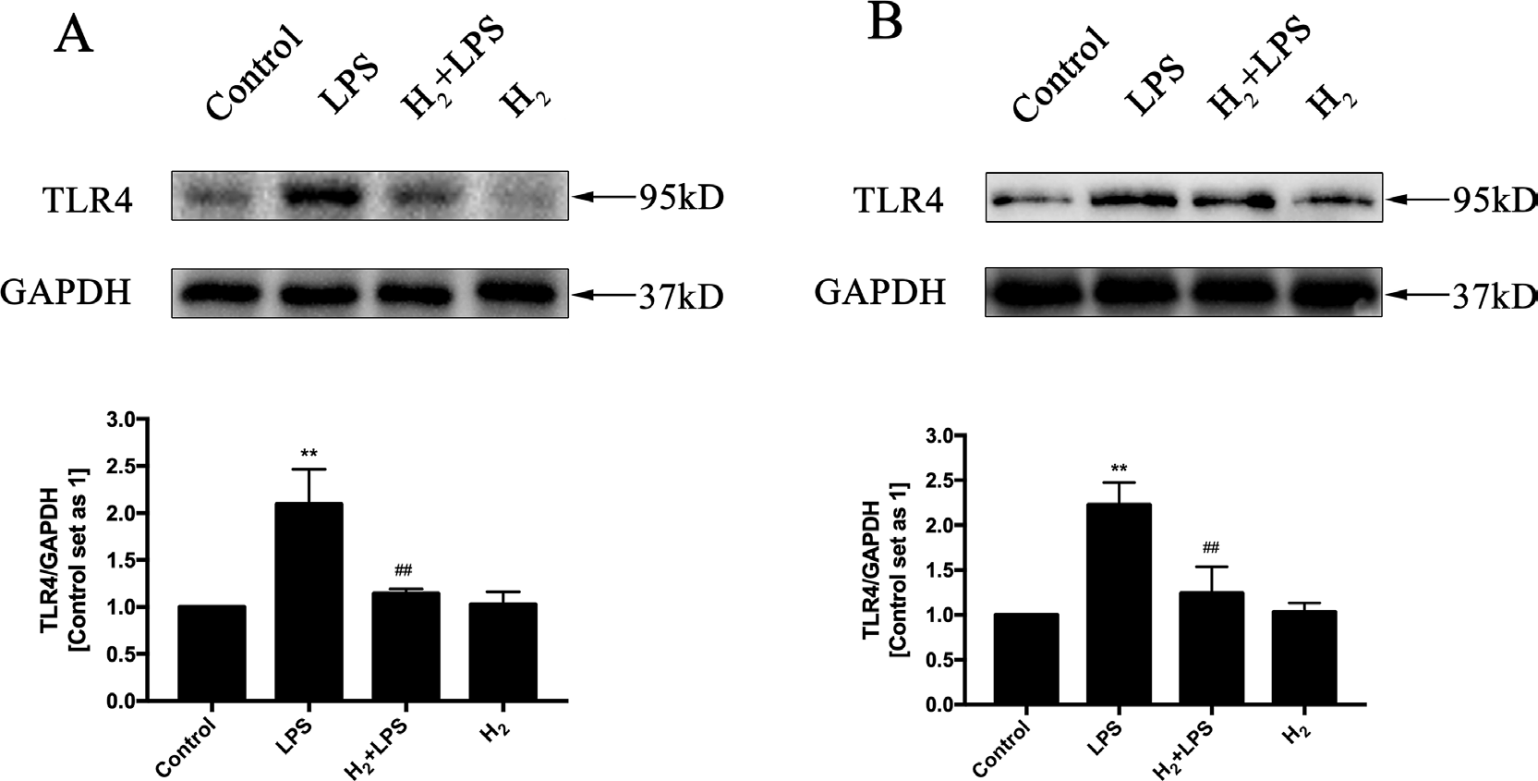
The effects of H_2_ on LPS-induced TLR4 expression. Representative Western blot and quantification of TLR4 to GAPDH *in vivo* **(A)** and *in vitro* **(B)**, respectively (*n* = 4). **p < 0.01 vs Control, ^##^ *p* < 0.01 vs LPS.

The increased cytokines previously discussed in LPS-challenged mice or in H9c2 cardiomyocytes are primarily induced by TLR4-mediated innate immune signaling, such as TRAF6-TAK1-IKKβ-NF-κB and TRAF6-MAPK-AP-1 signaling (Zhang et al., 2017a). Thus, we investigate whether the inhibition of H_2_ on LPS-induced inflammatory cytokines production is related to TLR4-mediated innate immune signaling in cardiomyocytes. The immunoblotting indicated that the increased phosphorylation of p38MAPK, JNK1/2, and ERK1/2 (**Figures 4A–F**) and the nuclear translocation of NF-κB (**Figures 5A, B**) by LPS were suppressed by H_2_ *in vivo* and *in vitro*. Moreover, the expression of TLR4 was increased after LPS stimulation, which was also blocked by H_2_ *in vivo* and *in vitro* (**Figures 6A, B**). Collectively, TLR4-mediated innate immune signaling in cardiomyocytes can be inhibited by H_2_ *in vivo* and *in vitro.*

## Discussion

The current studies were initiated to determine the effect of H_2_ intraperitoneal injection on LPS-induced cardiac dysfunction and its immune mechanism. We find that H_2_ protects against LPS-induced cardiac dysfunction *via* blocking TLR4 signaling-mediated cytokines expression.

Being traditionally recognized as a biologically inert gas, recent studies suggest that H_2_ can act as a biomolecule and has the potential ability to inhibit oxidative stress, inflammation, and apoptosis, thus, manifests cardioprotective effects or safeguards against tissue injury (Ostojic, 2017a; Ostojic, 2017b; Zhang et al., 2018). Supplements of exogenous H_2_ by inhalation or intraperitoneal injection of H_2_-rich saline attenuates myocardial ischemia/reperfusion (I/R) injury and improves cardiac function through anti-oxidative, anti-apoptotic, and anti-inflammatory effects (Hayashida et al., 2008; Sun et al., 2009; Zhang et al., 2011). Our previous studies indicated that H_2_ protects against isoproterenol (ISO)-induced cardiac dysfunction and cardiomyocyte hypertrophy *in vivo* or *in vitro* (Zhang et al., 2016a; Zhang et al., 2017b). *Akiko Noda* group indicated that chronic H_2_ inhalation prevents left ventricular hypertrophy in hypertensive Dahl salt-sensitive rats (Matsuoka et al., 2019). We reveled that H_2_ protects against LPS-induced cardiac dysfunction.

LPS (1 μg/ml) can upregulate pathologic hypertrophy marker ANP and BNP in H9c2 cardiomyocytes from 2 to 24 h and induce cellular hypertrophy in 3, 6, 12, and 24 h through calcineurin/NFAT-3 signaling pathway in H9c2 cardiomyocytes (Liu et al., 2008). In our study, H_2_-rich medium inhibits the upregulation of ANP and BNP by LPS *in vitro*. Left ventricular wall edema occurred during sepsis (Smeding et al., 2012; Castanares-Zapatero et al., 2013), and LPS induced enlargement in the cell size of cardiomyocytes *in vivo* (Wang et al., 2016b). In our study, left and right ventricular cavities were enlarged by LPS, LPS decreased the thickness of interventricular septum, and these phenotypes were improved by H_2_. The LV mass AW and LV mass AW (Corrected) were slight increased by LPS, and H_2_ decreases the upregulation of LV mass AW and LV mass AW (Corrected), although there were no statistical differences between groups. Drosatos et al. has also revealed that heart:body weight and lung:body weight ratios were not increased by LPS (Drosatos et al., 2013). The animal strains, animal sources, feeding environment, and the batch and dosage of LPS used might be responsible for these differences.

The endogenous H_2_ is produced by the bacterial species present in human gut, mouth and pharynx, the respiratory system, vagina, and skin (Ostojic, 2017b). Thus, given antibiotics for 4 days by mixing penicillin and streptomycin in the drinking water can reduce the H_2_ concentration below the 1-ppm detection limit in the expired air of male C57BL/6J mice, and the infarct size was significantly higher in mice administered with antibiotics than that in antibiotics non-treated mice (Shinbo et al., 2013). Another clinical investigation recently revealed overnight change in H_2_ concentration (ΔH_2_) was significantly lower in patients with chronic heart failure compared with controls and was positively correlated with cardiac index (Shibata et al., 2018). Ostojic also proposed an idea that an impaired production of endogenous H_2_ by intestinal microbiota might play a role in Parkinson disease pathogenesis (Ostojic, 2018). The key question that needs to be resolved is what is the function of the endogenous H_2_ in cardiovascular homeostasis and whether the endogenous H_2_ levels are related to the individual difference in the resistance of sepsis-induced cardiomyopathy.

LPS, a natural ligand for TLR4, induces cardiac inflammation and left ventricular dysfunction (Knuefermann et al., 2002; Drosatos et al., 2011; Drosatos et al., 2013). TLR4 also contributes to the development of cardiac remodeling following pressure overload, myocardial infarction, angiotensin II, or ISO infusion *in vivo* (Ha et al., 2005; Timmers et al., 2008; Dange et al., 2014; Dong et al., 2015; Han et al., 2017; Katare et al., 2017). Therefore, blocking TLR4-related innate immune signaling can improve the previously discussed stimuli-mediated cardiac dysfunction. H_2_ has been shown to improve LPS-induced lung (Qiu et al., 2011; Liang et al., 2012; Xie et al., 2012; Hattori et al., 2015; Liu et al., 2015; Dong et al., 2017), liver (Sun et al., 2011; Iketani et al., 2017), and bowel (Sakata et al., 2017) injuries. Herein, we showed that H_2_ inhibited LPS-mediated cardiac dysfunction, which can be induced by LPS and LPS-induced many kinds of cytokines, such as TNFα, IL-1β, and IL-18 (Thaik et al., 1995; Bryant et al., 1998; Li et al., 2002; Carlson et al., 2005; Suzuki et al., 2007; Liu et al., 2008; Wu et al., 2009; Chowdhury et al., 2013; Drosatos et al., 2013; Okuhara et al., 2017). These increased cytokines by LPS can be produced by inflammatory cells and by the cardiomyocytes and fibroblasts (Merx and Weber, 2007; Date et al., 2009; Avlas et al., 2011; Bai et al., 2016). In our study, we have shown that H_2_-rich medium reduced the mRNA levels of TNFα, IL-1β, and IL-18 in LPS-challenged H9c2 cardiomyocyte. The increased protein levels of TNFα, IL-1β, and IL-18 in the serum by LPS were also decreased after H_2_ pretreatment *in vivo*. Similar to our results, using different H_2_ delivery methods, *Atsunori Nakao* group and others have also indicated that H_2_ can decrease these pro-inflammatory cytokine levels in the target tissue or in serum in LPS-induced bowel dysfunction (LPS: 15 mg/kg) (Sakata et al., 2017) or acute lung injury [LPS: 25 μg/mouse (Xie et al., 2012) or 15 mg/kg (Liang et al., 2012)] or GalN/LPS-induced acute liver injury (LPS: 20 μg/kg) (Sun et al., 2011). However, if the dose of LPS increases to 30 mg/kg, H_2_ does not markedly ameliorate an elevation in inflammatory cytokines (Iketani et al., 2017). Therefore, the severity of disease is essential for the effects of H_2_; moreover, the dose of H_2_ used is also important.

It is well established that NF-κB and AP-1 are mainly responsible for TLR4 to induce the production of pro-inflammatory cytokines, such as TNFα, IL-1β, and IL-18 (Zhang et al., 2017a; Zhang et al., 2017c). The nuclear translocation of NF-κB and the phosphorylation of AP-1 upstream kinases ERK1/2, p38MAPK, and JNK by LPS in the heart are suppressed by H_2_. The suppression of H_2_ on NF-κB and MAPK exists widely in many kinds of animal models, such as isoproterenol-induced cardiac hypertrophy (Zhang et al., 2016a), LPS-induced acute lung injury (Xie et al., 2012), and intimal hyperplasia in arterialized vein grafts (Sun et al., 2012). Besides TLR4 signaling activation, LPS also enhanced TLR4 expression (Fallach et al., 2010), and the increased TLR4 was blocked by H_2_ in cardiomyocytes. However, the precise molecular targets of H_2_ in TLR4-mediated innate immune signaling still need further investigation.

## Conclusion

H_2_ protects against LPS-induced cardiac dysfunction *via* suppressing the excessive production of pro-inflammatory cytokines in the parenchymal cells of the heart by blocking TLR4-MAPKs/NF-κB signaling. Therefore, H_2_ is a promising natural agent for the prevention of LPS-induced cardiac dysfunction.

## Data Availability

The raw data supporting the conclusions of this manuscript will be made available by the authors, without undue reservation, to any qualified researcher.

## Ethics Statement

Male C57BL/6J mice (8 to 10 weeks of age) were used in the study. All animals were housed in a temperature-controlled animal facility with a 12-h light–dark cycle, and allowed to obtain rodent chow and water *ad libitum*. All animals received humane care in compliance with the Principles of Laboratory Animal Care formulated by the National Society of Medical Research and the Guide for the Care and Use of Laboratory Animals published by the NIH (8th Edition, Revised 2011) (Polhemus et al., 2017). The Institutional Animal Care and Use Committee (Zhongshan School of Medicine, Sun Yat-sen University) approved all animal procedures.

## Author Contributions

Conceived and designed the experiments: YZ. Drafted the manuscript: YZ and TW. Performed the experiments: ST, ZL, XH, YL, JX, XY, and YZ. Analyzed the data: ST and ZL. Contributed reagents/materials/analysis tools: JX and XY.

## Funding

This work was supported by the Natural Science Foundation of Guangdong Province (no. 2018A030313657) and the China Postdoctoral Science Foundation funded project (no. 2019M653238) to YZ and the National Natural Science Foundation of China (no. 81572585, no. 81372818) to TW.

## Conflict of Interest Statement

The authors declare that the research was conducted in the absence of any commercial or financial relationships that could be construed as a potential conflict of interest.
